# The incidence of hypersensitivity reactions to non-steroidal anti-inflammatory drugs in patients with chronic urticaria

**DOI:** 10.1186/2045-7022-4-S3-P127

**Published:** 2014-07-18

**Authors:** Pavel Kolkhir, Olga Olisova, Nikolay Kochergin, Olga Kosoukhova

**Affiliations:** 1 First Moscow State Medical University, Department of Dermatology and Venereology, Russia

## Background

According to recent studies cutaneous manifestations to non-steroidal anti-inflammatory drugs (NSAID) may affect 0.3% of general population and the prevalence of aspirin hypersensitivity is as high as 27-35% in patients with chronic urticaria (CU). NSAID may be the cause of acute urticaria but in CU patients it usually exacerbates the disease by inhibition of the cyclooxygenase, which results in an increased synthesis and release of cysteinyl leukotrienes. The aim of our study was to investigate the incidence of reactions to NSAID as well as a possible relationship between NSAID hypersensitivity and the quality of life, disease activity and the response to treatment in CU patients.

## Method

We observed 110 patients with CU of different severity. Depending on the presence of reactions after NSAID intake according to medical history and a drug provocation test (DPT), CU patients were divided into two subgroups: positive (n=25, 22.7%) and negative (n=85, 77.3%).

## Results

The decline in the quality of life appeared to be more sharp in the subgroup of NSAID-positive patients than in NSAID-negative group (mean±SD: 55.9±10.9 vs 65.2±9.7%; p=0.008). Most of NSAID-positive patients had multiple NSAID hypersensitivity (n=15, 60%), a severe disease course (n=17, 68%) and a poor response to treatment with antihistamines (n=13, 52%).

## Conclusions

NSAID reactions may develop in about ¼ of CU patients. DPT with aspirin is a "gold standard" which allows to confirm the diagnosis of NSAID-induced urticaria/angioedema in more than 90% of cases with a corresponding history. In this case the use of the drug should be avoided and the safety of an alternative drug should be confirmed by oral challenge. Reactions to NSAID are often observed in CU patients with the low quality of life, severe disease course and poor response to antihistamine treatment. In such patients, leukotriene receptor antagonists taken alone or in addition to antihistamines may have some benefit.

